# Effect of PCSK9 on atherosclerotic cardiovascular diseases and its mechanisms: Focus on immune regulation

**DOI:** 10.3389/fcvm.2023.1148486

**Published:** 2023-03-10

**Authors:** Minglu Ma, Chang Hou, Jian Liu

**Affiliations:** ^1^ Department of Cardiology, Peking University People's Hospital, Beijing, China; ^2^ Beijing Key Laboratory of Early Prediction and Intervention of Acute Myocardial Infarction, Peking University People's Hospital, Beijing, China

**Keywords:** PCSK9, atherosclerotic cardiovascular diseases, functional mechanism, immune regulation, low-density lipoprotein-cholesterol

## Abstract

Atherosclerosis is a basic pathological characteristic of many cardiovascular diseases, and if not effectively treated, patients with such disease may progress to atherosclerotic cardiovascular diseases (ASCVDs) and even heart failure. The level of plasma proprotein convertase subtilisin/kexin type 9 (PCSK9) is significantly higher in patients with ASCVDs than in the healthy population, suggesting that it may be a promising new target for the treatment of ASCVDs. PCSK9 produced by the liver and released into circulation inhibits the clearance of plasma low-density lipoprotein-cholesterol (LDL-C), mainly by downregulating the level of LDL-C receptor (LDLR) on the surface of hepatocytes, leading to upregulated LDL-C in plasma. Numerous studies have revealed that PCSK9 may cause poor prognosis of ASCVDs by activating the inflammatory response and promoting the process of thrombosis and cell death independent of its lipid-regulatory function, yet the underlying mechanisms still need to be further clarified. In patients with ASCVDs who are intolerant to statins or whose plasma LDL-C levels fail to descend to the target value after treatment with high-dose statins, PCSK9 inhibitors often improve their clinical outcomes. Here, we summarize the biological characteristics and functional mechanisms of PCSK9, highlighting its immunoregulatory function. We also discuss the effects of PCSK9 on common ASCVDs.

## 1. Introduction

Atherosclerosis is an early pathological change in many cardiovascular diseases (1). The occurrence and progression of atherosclerosis and atherosclerotic cardiovascular diseases (ASCVDs) are associated with hyperlipidemia but also involves an imbalance between proinflammatory and anti-inflammatory response (2, 3). During this process, the injured arterial endothelium, multiple immune cells (such as macrophages, dendritic cells, B cells, T cells, and mast cells), macrophage-like vascular smooth muscle cells (VSMCs), adhesion molecules, cytokines, and oxidized low-density lipoprotein (ox-LDL) deposited beneath the vascular intima work together to activate the local immune response, while foam cells always have moderate proinflammatory functions and mainly participate in the formation of the lipid core (3–6). Through earlier detection, earlier diagnosis, and more effective treatment, the occurrence and progression of atherosclerosis and ASCVDs can be better controlled.

Statins, as a first-line drug recommended by clinical guidelines for the treatment of atherosclerosis and ASCVDs, inhibits cholesterol synthesis by inhibiting the activation of the rate-limiting enzyme 3-hydroxy-3-methylglutaryl-coenzyme A (HMG-CoA) reductase, thereby reducing cholesterol concentrations in the endoplasmic reticulum (ER) (7, 8). Statin resistance occurs mainly because the low cholesterol concentration in the ER promotes the activation of sterol regulatory element-binding protein 2 (SREBP2) and the subsequent production of PCSK9 (9). PCSK9 inhibitors have been shown to be beneficial in the treatment of patients with atherosclerosis and ASCVDs (10–15).

PCSK9, also known as neural apoptosis-regulated convertase-1 (NARC-1), was first identified in 2003 (16). As the ninth and last member of the proteinase K subfamily of subtilases, PCSK9 undergoes self-cleavage and multiple rounds of post-translational modifications before becoming the mature form (17–19). PCSK9 functions in a nonenzymatic fashion, which differs from other family members (20, 21). The carboxy-terminal domain (residues 452 to 692) of full-length PCSK9 protein shares structural homology with the resistin, which is essential for the formation of PCSK9-low-density lipoprotein receptor (LDLR) dimer (22). The critical role of PCSK9 in cholesterol regulation was discovered, as its gain-of-function variants could lead to human familial hypercholesterolemia, while its nonsense mutations (Y142X and C679X) in African Americans were associated with 40% lower plasma level of low-density lipoprotein-cholesterol (LDL-C) and lower risk of coronary heart disease (CHD) (23, 24). Then, accumulating evidence has suggested that the effects of PCSK9 on ASCVDs may be associated with immunoregulation, platelet activation, thrombosis, and multiple forms of cell death, which may be independent of its lipid-lowering capacity (25–27). In some clinical trials, the correlation between PCSK9 and immune response has been demonstrated in patients with atherosclerotic disease, coronary artery disease, systemic lupus erythematosus, and human immunodeficiency virus (HIV)-infected (28–31). Experiments *in vitro* and in mice further support the immunoregulatory function of PCSK9 (32–35).

Here, we used the following keywords to filter corresponding papers in PubMed: “PCSK9 and atherosclerosis”, “PCSK9 and myocardial infarction”, “PCSK9 and inflammation”, “PCSK9 and platelets”, “PCSK9 and autophagy”, “PCSK9 and apoptosis”, as well as “PCSK9 and pyroptosis”. We first introduce the production of PCSK9 and the molecular mechanisms that regulate its expression. We then summarize the functional mechanisms of PCSK9 and its inhibitors in the cardiovascular system, such as the regulatory function in lipid metabolism, immune response, thrombosis, and multiple modes of cell death, with a particular focus on its immunoregulation. Subsequently, we show the effect of PCSK9 and its inhibitors on the prognosis of atherosclerotic and myocardial infarction patients. Finally, we propose several issues that need to be addressed in the future regarding PCSK9 and cardiovascular diseases.

## 2. Regulation of PCSK9 production and expression

The liver is the major organ that produces PCSK9, and the kidney, the small intestine, the pancreas, the lung, and the central nervous system also produce small amounts of PCSK9 (16, 36). Under physiological conditions, the expression of PCSK9 was detected in cultured human smooth muscle cells (SMCs), while was undetectable in human umbilical vein endothelial cells (HUVECs), monocytes and macrophages (37). Under an inflammatory state [lipopolysaccharide (LPS) treatment], HUVECs can produce PCSK9 (38). When atherosclerosis occurs, various types of cells, especially SMCs, endothelial cells and macrophages in the injured vessels produce large amounts of PCSK9 at the transcriptional and translational levels in response to the stimulation of low shear stress, lipopolysaccharide (LPS), tumor necrosis factor-alpha (TNF-α), interleukin-1beta (IL-1β), ox-LDL, reactive oxygen species (ROS), and mitochondrial DNA (mtDNA) and mitochondria-derived reactive oxygen species (mtROS) released from damaged mitochondria in ruptured cells (37, 39–42). When myocardial infarction occurs, the expression level of PCSK9 is also upregulated in the ischemic heart tissue, mostly in the border zone, and hypoxia as well as proinflammatory cytokines may be the key factors that upregulate its expression (43, 44).

**Figure 1:**
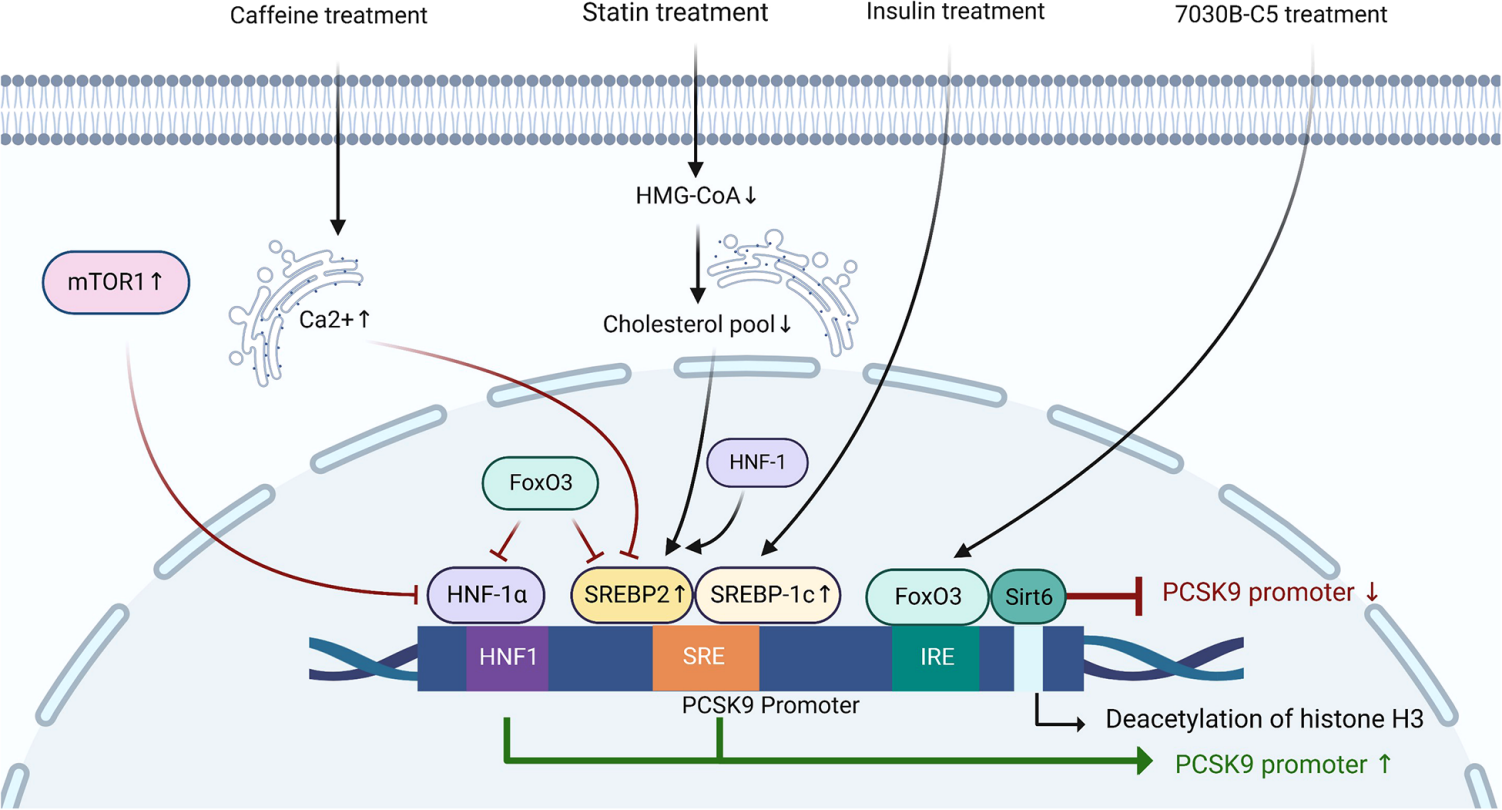
The regulation of PCSK9 expression at the transcriptional level. HNF1, hepatocyte nuclear factor-1; SRE, sterol-regulatory element; SREBP, sterol regulatory element-binding protein; IRE, insulin-response element; FoxO3, forkhead box O3; Sirt6, sirtuin-6; mTOR1, mechanistic target of rapamycin complex 1; HMG-CoA, 3-hydroxy-3-methyl-glutaryl-coenzyme A.

The expression of PCSK9 is mainly regulated by SREBP2, hepatocyte nuclear factor-1α (HNF-1α), and forkhead box O3 (FoxO3) at the transcriptional level (45–48). Sequence analysis of the 5′ flanking region from −2,112 to −94 of the PCSK9 gene revealed the presence of the sterol regulatory element (SRE) site (5′-GTGGCGTGAT-3′) in the proximal region of the PCSK9 promoter (49, 50). In the process of sterol-dependent transcriptional regulation of PCSK9, the SRE site and the adjacent upstream nucleotides are critically required (45). The SRE site is the target site for SREBPs (45). After treatment with statins, decreased cholesterol concentrations in the ER promote the production of PCSK9 by activating SREBP2, leading to a weak effect of statin treatment in some ASCVD patients (49). The transcriptional activation of PCSK9 induced by insulin is dependent on SREBP1c (50). Caffeine inhibits the expression of SREBP2 at the transcriptional level by increasing the Ca^2+^ concentration in the hepatic ER, thereby reducing the expression of PCSK9 and the risk of cardiovascular disease (51). Besides, both HNF1α and HNF1β are positive regulators of PCSK9 transcription, although there is little literature mentioning the role of HNF1β in this process (52). HNF1α regulates the transcription of PCSK9 through hepatocyte nuclear factor 1 (HNF1) site located 28 bp upstream from SRE (53). Mutation of the HNF1 site significantly inhibits the activity of PCSK9 promoter, which is dependent on both its direct effect and its indirect effect of inhibiting the activity of SRE site (46). In mice, the activated mechanistic target of rapamycin complex 1 (mTOR1) pathway suppresses the transcription of PCSK9 by silencing HNF-1α (54). FoxO3 is a negative regulator of PCSK9 transcription (47). Sirtuin-6 (Sirt6) is an NAD + -dependent histone deacetylase (55). After the interaction of FoxO3 with insulin-response element (IRE), Sirt6 binds to the PCSK9 promoter to deacetylate histone H3 at lysines 9 and 56, resulting in the attenuated activity of PCSK9 promoter (47, 56). FoxO3 and Sirt6 also suppress the transcriptional activity of SRE and HNF1 (47). Understanding the molecular mechanisms that regulate the production of PCSK9 is of great value to effectively inhibit the overexpression of PCSK9 and alleviate the risk of ASCVDs (Figure 1).

## 3. Molecular functions of PCSK9 and its mechanisms

The occurrence and development of atherosclerosis is associated with abnormal lipid metabolism and excessive proinflammatory response (57, 58). Platelet activation and thrombosis secondary to coronary atherosclerotic plaque rupture can lead to myocardial infarction, a common disorder in ASCVDs (59). Subsequently, cardiomyocytes die due to ischemia and hypoxia (59). Here, we describe the molecular functions of PCSK9 and its mechanisms around the above points.

### 3.1. Effect of PCSK9 on lipid metabolism

**Figure 2:**
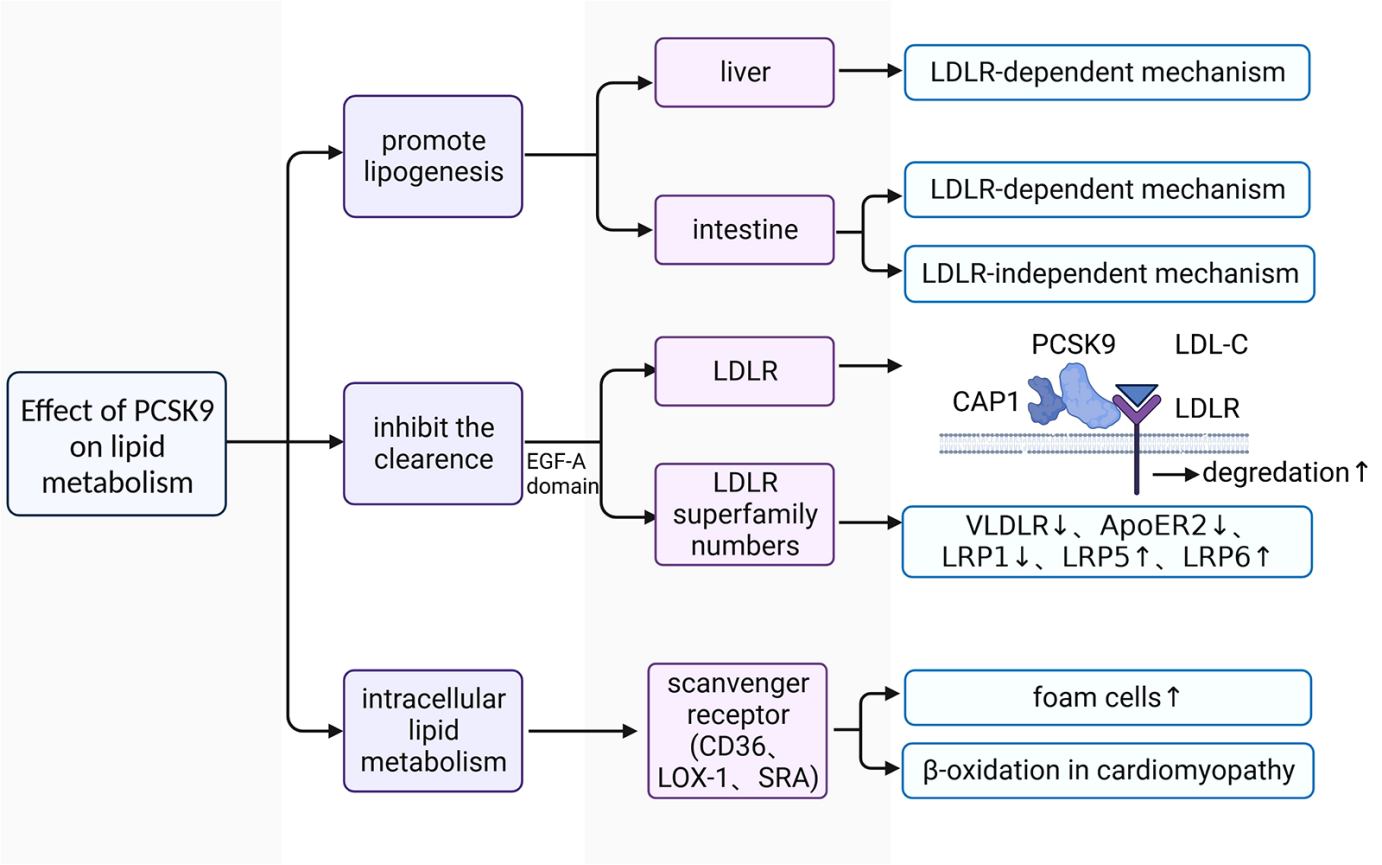
The functional mechanism of PCSK9 in regulating lipid metabolism. LDL-C, low-density lipoprotein-cholesterol; LDLR, low-density lipoprotein receptor; VLDLR, very low-density lipoprotein receptor; ApoER2, apolipoprotein E receptor 2; PCSK9, proprotein convertase subtilisin/kexin type 9; CAP1, cyclase associated protein 1; LOX1, lectin-like oxidized low-density lipoprotein receptor 1; SRA, scavenger receptor type A; EGF-A, epidermal growth factor precursor homology domain A; LRP1, LDLR-related protein 1; LRP5, LDLR-related protein 5; LRP6, LDLR-related protein 6.

The plasma levels of LDL-C are closely associated with an increased risk of ASCVDs (60). Downregulating LDL-C concentrations can help to reduce the incidence of adverse cardiovascular events (61, 62). Approximately 60%–70% of plasma LDL-C is cleared in the liver after binding to LDLR on the surface of hepatocytes (63). LDL-C-lowering LDLR variants are associated with lower risk of CHD (64). PCSK9 affects the plasma lipid and lipoprotein levels mainly by downregulating LDLR in the liver (65). It has been confirmed that gain-of-function mutations of PCSK9 lead to increased risks of hyperlipidemia and ASCVDs, whereas loss-of-function mutations of PCSK9 reduced plasma levels of LDL-C and ASCVDs risk (Figure 2) (66–68).

PCSK9 promotes the synthesis of lipoprotein (69–72). In C57BL/6 wild-type (WT) and LDLR-/- mice with high fat diet (HFD), researchers found that human (h) PCSK9 promotes the synthesis and secretion of cholesterol and triglycerides in the intestine in both mice, but only significantly increased the expression of key genes involved in lipogenesis at the transcriptional and translational levels in the liver in WT mice (70, 72). These findings indicate that PCSK9 promotes lipoprotein production in the liver only through LDLR-dependent mechanisms, whereas its facilitation of lipid production is achieved through both LDLR-dependent and LDLR-independent mechanisms in the intestine (70, 72).

PCSK9 promotes the upregulation of plasma lipoprotein *via* reducing their removal. LDLR is a key receptor for PCSK9 to regulate lipid metabolism (73). Under physiological conditions, plasma LDL-C binds to LDLR on the surface of hepatocytes to form the LDL-C-LDLR dimer, which is then transferred into the intracellular space (74). LDL-C is then separated from LDLR in endosomes and degraded in lysosomes (74). Subsequently, the intracellular free LDLR is recycled to the cell surface to participate in a new round of LDL-C transport and degradation (74). Under pathological conditions, the liver produces large amounts of PCSK9 and releases it into the plasma. The increased PCSK9 conjunct with cyclase associated protein 1 (CAP-1) binds to the LDL-C-LDLR dimer and promotes its degradation in lysosomes through a caveolin-dependent mechanism, leading to the downregulation of LDLR on the surface of hepatocytes (75). Some LDL-C-LDLR dimers that are bound to free PCSK9 without CAP-1 are endocytosed and transferred to endosomes through a clathrin-dependent mechanism, and LDLR is then released from the dimer and recycled to the cell surface without the diminution of total LDLR (75). CAP-1 is essential for PCSK9 to regulate lipid metabolism (75). There are also other LDLR family members involved in the lipid regulatory process of PCSK9, such as very low-density lipoprotein receptor (VLDLR), apolipoprotein E receptor 2 (ApoER2), LDLR-related protein 1 (LRP1), LDLR-related protein 5 (LRP5), and LDLR-related protein 6 (LRP6) (76–78). Because VLDLR and ApoER2 share a common epidermal growth factor precursor homology domain A (EGF-A) with LDLR, PCSK9 acts on the EGF-A domain of both receptors and promotes their degradation in the same manner as LDLR (79). PCSK9 is able to mediate the degradation of LRP1 (77). LRP5 and LRP6 act as coreceptors of Wnt ligands and activate Wnt-related signaling (80). Recently, much attention has been paid to the interaction between PCSK9 and LRP5/6 in the development and progression of atherosclerosis (78, 81). LRP5 is capable for promoting the accumulation of cholesterol in macrophages and the formation of foam cells (78). There is a positive feedback effect between LRP5 expression and the level of plasma PCSK9 (78, 82). Despite the lack of direct evidence, LRPs interact with PCSK9 may also rely on the epidermal growth factor-like (EGF-like) domain as it is shared in these LDLR superfamily members (83).

In addition, PCSK9 is also involved in intracellular lipid metabolism. After stimulation with recombinant PCSK9, the expression levels of lectin-like oxidized low-density lipoprotein receptor 1 (LOX-1), class A scavenger receptor (SRA), and CD36 on the surface of macrophages are 2- to 5-fold higher than those of unstimulated macrophages, and the uptake of ox-LDL is also increased by approximately 5-fold (84). LOX-1 is a key receptor for macrophages to engulf ox-LDL (38). There is a positive feedback between LOX-1 and PCSK9, promoting the transformation of macrophages and VSMCs into foam cells (38). PCSK9 also inhibits the expression of ATP-binding cassette transporters [such as (ATP-binding cassette transporter A1) ABCA1] on macrophages, which are key transporters for cholesterol efflux (85). Thus, PCSK9 promotes the formation of foam cells from two different aspects. PCSK9 also affects lipid metabolism in cardiomyocytes. Cardiomyocytes meet their own energy requirement mainly through β-oxidation of fatty acids (FAs), and reactive oxygen species (ROS) is usually produced in this process (86). When oxidative stress occurs, excess cytotoxic ROS results in nonspecific oxidation of proteins, lipids, and DNA (86). The major route that FAs enter cardiomyocytes is active transport, which is mainly mediated by CD36, with fatty acid transporter (FATP) and fatty acid binding protein (FABP) involved (87).

Importantly, it is not beneficial to completely remove PCSK9 *in vivo* (88). Complete knockout of PCSK9 in mice is deleterious, even leading to heart failure with preserved ejection fraction (HFpEF) (88). The pathological deposition of FAs in cardiomyocytes causes lipotoxicity, with a reduced density of mitochondrial cristae (88). Therefore, it is necessary to control the dosage of PCSK9 inhibitors in a reasonable range in the treatment of ASCVDs. Fortunately, in large clinical trials, PCSK9 inhibitors are tolerated and effectively, without the increased risk of heart failure (14, 89).

### 3.2. Role of PCSK9 in the inflammatory response

**Figure 3:**
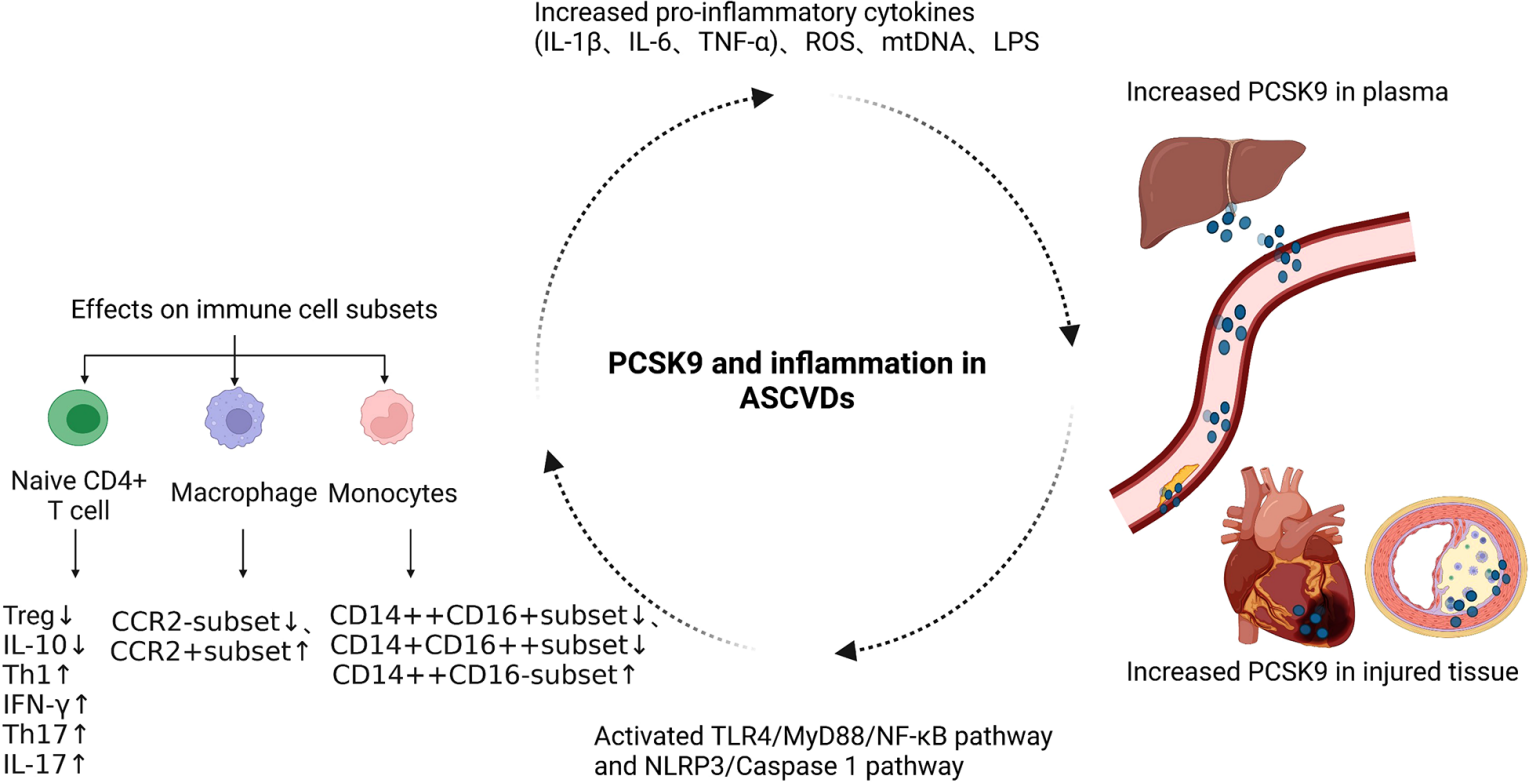
The functional mechanism of PCSK9 in regulating the immune response of ASCVDs. ASCVDs, atherosclerotic cardiovascular diseases; NLRP3, NOD-like receptor family pyrin domain containing 3; TLR4, Toll-Like Receptor 4; ROS, reactive oxygen species; mtDNA, mitochondrial DNA; LPS, lipopolysaccharide.

In recent years, the functional pleiotropy of PCSK9 has been gradually recognized, and its immunoregulatory function has attracted much attention, especially in the fields of autoimmune diseases, cancer, and cardiovascular diseases (25, 29, 90). PCSK9 accelerates the development of ASCVDs through its proinflammatory function independent of its effect on lipid, but the molecular mechanisms by which PCSK9 regulates the immune response are still not well elucidated (Figure 3) (40, 91).

Several clinical studies have confirmed that PCSK9 promotes the progression of ASCVDs by its proinflammatory function. In the Further Cardiovascular Outcomes Research with PCSK9 Inhibition in Subjects with Elevated Risk (FOURIER) trial, 27,564 patients with stable ASCVDs and LDL-C ≥ 70 mg/dl were randomly assigned to the evolocumab group and placebo group, both of which were then divided into three subgroups based on the level of high sensitivity C-reactive protein (hs-CRP) (<1, 1–3, and >3 mg/dl) at baseline (10, 92). Patients with higher hs-CRP had a greater risk of the primary and key secondary endpoint events, showing that inflammation may be an independent risk factor in ASCVDs (90). In patients with higher hs-CRP, the reduction in the absolute risk was more significant in the evolocumab group than in the placebo group (93). Thus, the protective effect of evolocumab on ASCVD patients, at least in part, relies on its anti-inflammatory function. In addition, in the ODYSSEY OUTCOMES trial, alirocumab reduced plasma lipoprotein(a) and the risk of major adverse cardiac events (MACEs) in patients with acute coronary syndrome (ACS) (94). Lipoprotein(a) is a biomarker positively correlated with inflammatory response (95). In the PACMAN-AMI trial, the use of alirocumab in addition to high-intensity statin therapy resulted in a significant regression of percent atheroma volume in the two non-infarct-related coronary arteries and increased plaque stability in patients with acute myocardial infarction without a reduction of hs-CRP (14). However, further research revealed that alirocumab caused a greater reduction in the mean angular extension of macrophages, indicating that the local inflammatory response was decreased in this process (14). Therefore, it may not be sufficient to select hs-CRP as the only biomarker reflecting the intensity of the inflammatory response, and PCSK9 inhibitors are useful to inhibit the local inflammatory response in ASCVD patients (14, 96). Moreover, PCSK9 inhibitors suppress the accumulation of the proinflammatory cytokine IL-6 in the plasma of stable coronary artery disease patients with the IL6-74CC genotype and high level of lipoprotein(a) (97). However, in patients with elevated lipoprotein(a) and increased cardiovascular risk, evolocumab only modestly reduces the lipoprotein(a), and residual high level of lipoprotein(a) leads to persistent arterial wall inflammation (98). Thus, whether PCSK9 inhibitors have anti-inflammatory function and the mechanisms involved need to be further explored.

In addition to regulating inflammatory cytokines, numerous studies have linked PCSK9 with immune cell subsets in ASCVDs. Circulating monocytes are usually divided into three categories, including classical monocytes (CD14++CD16−; CMs), intermediate monocytes (CD14++CD16+; IMs) and nonclassical monocytes (CD14+CD16++; NCMs), among which CMs have strong proinflammatory functions (99, 100). When stable coronary artery disease occurs, the proportion of CMs to total monocytes is larger in patients with higher PCSK9 levels. Increased PCSK9 promotes the polarization of monocytes from IM and NCM-like phenotypes to a CM-like phenotype (101). In patients with hyperlipidemia, PCSK9 induces the expression of C-C chemokine receptor type 2 (CCR2) on the surface of monocytes and enhances their migration ability (102).

Previous studies have shown that PCSK9 siRNA can inhibit ox-LDL-induced proinflammatory function of THP-1-derived macrophages *via* suppressing the activation of the NF-κB pathway (35). Compared with C57BL6/J WT mice, the C57BL6/J PCSK9 knockout (KO) mice have decreased infarct size and improved cardiac function due to inhibition of the polarization of M1-type macrophages, and the suppression of the TLR4/MyD88/NF-κB pathway may be involved in this process, which is consistent with the above results (43). In apolipoprotein E (apoE) KO mice with hyperlipidemia-induced atherosclerosis, PCSK9 silencing inhibits the progression of plaque volume, the accumulation of macrophages in lesion areas, and the secretion of inflammatory cytokines, such as TNF-α and IL-1β, by macrophages, which is accompanied by limited intracellular activation of the TLR4/NF-κB pathway (32). Because the AT04A anti-PCSK9 vaccine reduces the expression of NLR family pyrin domain containing 3 (NLRP3) and proinflammatory biomarkers in macrophages, both the NLRP3 and TLR4/NF-κB pathways may be involved in the PCSK9-mediated regulation of macrophage function (32).

The adaptive immune response is also found to participate in the later stage of atherosclerosis as T cells localize near the ruptured areas of unstable plaques in ischemic heart tissue (103). Increased PCSK9 promotes the maturation of dendritic cells and the differentiation of naive CD4+ T cells toward the Th1 and Th17 subsets, resulting in increased secretion of interferon-γ (IFN-γ) and interleukin 17A (IL-17A) (104). PCSK9 inhibitors promote the differentiation of naive CD4+ T cells into regulatory T cells (Tregs) and the production of anti-inflammatory cytokines, such as interleukin 10 (IL-10) and transforming growth factor beta (TGF-β), which contribute to the resolution of inflammation and good prognosis of ASCVDs (104). Apart from the cardiovascular system, PCSK9 also regulates the adaptive immune response in the tumor microenvironment. The interaction between LDLR and T cell receptor (TCR) regulates TCR recycling and signaling, thus promoting the differentiation of CD8+ T cells into cytotoxic T lymphocytes (90). PCSK9 inhibits the killing function of CD8+ T cells *via* binding to LDLR and preventing the recycling of LDLR-TCR complex to the plasma membrane (90). Thus, we postulate that PCSK9/LDLR may also be a significant target for regulating the adaptive immune response in ASCVDs, but this specific mechanism has not been confirmed.

### 3.3. Impact of PCSK9 on platelet activation and thrombosis

Under pathological conditions, such as hyperlipidemia, hyperglycemia, and atherosclerosis, high LDL-C levels are associated with enhanced platelet reactivity and thromboxane production (105, 106). Many factors are involved in the regulation of this process, which is still not completely clear (107). CD36 is associated with platelet reactivity, activation and thrombosis under hyperlipidemic conditions (108). During the processes of plaque formation with inflammation and phospholipid oxidation, LDL is converted to ox-LDL (109). Ox-LDL and hyperlipidemia activate blood platelets *via* a CD36 mediated pathway (110, 111). In patients with familial hypercholesterolemia, researchers found that ox-LDL induced the activation of platelets *via* the activation of CD36, LOX-1, and NADPH oxidase 2 (NOX2) (112). PCSK9 is a positive modulator in this process (113). Recently, researchers have found that PCSK9 promotes the aggregation, activation, spreading of platelets and thrombosis by interacting with CD36 on its surface and activating the downstream p38 mitogen-activated protein kinase (MAPK)/cytosolic phospholipase A2/cyclooxygenase-1/thromboxane A2 pathway (26, 113). In C57BL/6J WT mice, PCSK9 injection promotes FeCl3-induced mesenteric artery thrombosis through binding to CD36 receptor in platelets (26). When myocardial infarction occurs, PCSK9 promotes the generation of ROS and activates CD36 in platelets, resulting in microvascular obstruction and enlarged infarct size (26). Therefore, the experimental results mentioned above confirm the positive role of PCSK9 in platelet activation and thrombosis, which may be adverse in ischemic heart disease.

### 3.4. Influence of PCSK9 on apoptosis, autophagy, and pyroptosis

In the development of atherosclerosis, ox-LDL is one of the important factors causing the dysfunction of endothelial cells and the primary factor promoting apoptosis in endothelial cells by upregulating the apoptosis-related factors, Bcl2-associated X (Bax), caspase 3, and caspase 9, as well as downregulating the antiapoptotic factor, Bcl2 (114, 115). PCSK9 is a key mediator of ox-LDL-induced apoptosis in HUVECs (116). The proapoptotic function of ox-LDL mainly depends on the upregulation of PCSK9 expression and the activation of its downstream MAPK signaling pathway, especially the phosphorylation of c-Jun N-terminal kinase (JNK) and p38 (116). Upregulated PCSK9 promotes the apoptosis of endothelial cells in atherosclerotic lesions (116). Targeting PCSK9 with short hairpin RNA (shRNA)-PCSK9 inhibits the phosphorylation of p38 and JNK induced by ox-LDL, as well as downregulates the ratio of Bax to Bcl2, thus repressing the apoptosis of endothelial cells (116).

PCSK9 is associated with autophagy (44). Autophagy removes damaged mitochondria, which is of great benefit to maintain cell survival and normal function (117). MtDNA that escapes from autophagy leads to inflammation and heart failure (118). When 3-methyladenine is used to inhibit autophagy, PCSK9 accumulates in the cytoplasm, suggesting that it may be associated with autophagy (41). Under inflammatory conditions, increased PCSK9 destroys mtDNA and promotes the formation of mtROS in SMCs, which further promotes the upregulation of PCSK9 and LOX-1 (41). When myocardial infarction develops in mice with PCSK9 knockdown or with PCSK9 inhibitor Pep2-8 treatment, the infarct size is smaller and the autophagy is reduced compared with WT mice (44).

During the process of chronic myocardial ischemia, upregulated PCSK9 induces mtDNA damage, which activates NLRP3 inflammasome signaling [NLRP3, apoptosis-associated speck-like protein containing a caspase recruitment domain (ASC), caspase 1, IL-1β, and interleukin-18 (IL-18)] and promotes caspase 1-dependent pyroptosis (27). The pyroptosis marker, N-terminal gasdermin D fragment (GSDMD-NT), is highly expressed in the peripheral area of the infarct zone (27, 119). In PCSK9 knockout mice, the activation of NLRP3 and the upregulation of GSDMD-NT in the ischemic heart are significantly inhibited (27).

In summary, PCSK9 inhibits autophagy but promotes apoptosis and pyroptosis, which is unfavorable for the prognosis of ASCVDs.

## 4. Relationship of PCSK9 with ASCVDs

### 4.1. PCSK9 and atherosclerosis

There is a significant positive correlation between plasma PCSK9 levels and the risk of atherosclerosis. Feeding a high-fat diet to mice transduced with an adeno-associated virus overexpressing the PCSK9 gene induces hypercholesterolemia and even atherosclerosis (120). Similarly, transgenic pigs with the D374Y gain-of-function mutation in the PCSK9 gene are more prone to develop atherosclerosis than WT pigs when fed a high-fat and high-cholesterol diet (121). In patients with rheumatoid arthritis (RA), the plasma level of PCSK9 and the ratio of PCSK9 to LDLR are positively correlated with the occurrence and development of atherosclerosis (122). LRP5 and LRP6, as coreceptors of PCSK9, promote atherosclerosis by activating the Wnt/β-catenin signaling pathway, resulting in significant proliferation of VSMCs and decreased anti-inflammatory macrophages (78, 81). However, there may still be lack of correlation between the plasma PCSK9 level and the severity of subclinical atherosclerosis in patients without symptoms of cardiovascular diseases (123).

Researchers have found that LincRNA-p21 binding to miR-221 promotes the deacetylation of PCSK9 *via* negatively regulating the expression of SIRT1, eventually leading to the enhance of the proliferation, migration and angiogenesis of arterial endothelial cells, ultimately diminishing the development of atherosclerosis (124). Treatment of ApoE-/- mice on a high-fat diet with berberine reverses the progression of atherosclerotic plaques by downregulating PCSK9 expression and upregulating LDLR expression through the activation of the ERK1/2 signaling pathway in hepatocytes (125). Similarly, in ApoE-/- mice with a high-cholesterol diet (1.25% w/w), the sirtuin 1 activator [SRT3025 and 20(S)-protopanaxadiol] exerts an anti-atherosclerotic function by reducing plasma PCSK9 and upregulating LDLR (126, 127).

The proatherogenic effect of PCSK9 may be independent of its lipid-regulatory function (32). In ApoE-/- mice fed a high-fat diet, the overexpression of PCSK9 promotes the progression of atherosclerotic plaques without upregulating the plasma cholesterol level (32). The increased PCSK9 could accelerate atherosclerosis through activating the TLR4/NF-κB signaling pathway and promoting inflammation (32). In homocysteine-treated ApoE-/- mice with a methionine diet, upregulated PCSK9 inhibits cholesterol efflux mediated by ABCA1 and ABCG1 in macrophages, thereby accelerating the formation of foam cells (128). Therefore, PCSK9 promotes the development of atherosclerosis dependent on inflammatory regulation in part.

### 4.2. PCSK9 and myocardial infarction

Myocardial infarction is one of the major causes of mortality worldwide (129). Patients with myocardial infarction have a high risk of ischemia reperfusion injury and the “no-reflow” phenomenon after successful percutaneous coronary intervention treatment, and more improvement in therapeutic strategies is still needed (129, 130). Cardiomyocytes from adult mice express PCSK9 at both the transcriptional and translational levels (131). One week after ligation of the left anterior descending coronary artery in C57BL/6J WT mice, the increased expression of PCSK9 in the zone bordering the infarct area leads to the progression of myocardial infarction (44). Non-ST-segment elevation myocardial infarction patients with higher plasma levels of PCSK9 have a higher risk of MACEs than those with moderate or low levels of PCSK9 (132). In young males with myocardial infarction, higher level PCSK9 exacerbates the severity of coronary artery diseases and increases the risk of MACEs (133). Consistently, patients in the Chinese Han population with the PCSK9 R93C variant (PCSK9 loss-of-function mutant) have a lower risk of myocardial infarction (134). Therefore, the level of plasma PCSK9 is positively related to the occurrence risk and the severity of myocardial infarction.

PCSK9 inhibitors can further reduce plasma LDL-C in addition to statins treatment at the maximum dose, which is beneficial to the prognosis of myocardial infarction (135). In the PACMAN-AMI study, 300 patients with acute myocardial infarction treated with rosuvastatin (20 mg/day) were randomly divided into two groups. Patients in the experimental group received alirocumab (150 mg) subcutaneously once every 2 weeks, and those in the control group received an equal dose of placebo. Fifty-two weeks after the initial treatment, intravascular ultrasonography, near-infrared spectroscopy, and optical coherence tomography results all showed that the use of alirocumab significantly reversed the plaque in the two non-infarct-related coronary arteries and improved the stabilization of plaques (14, 136). In the FOURIER study, the reduction of absolute occurrence risk of the endpoint events after using evolocumab in stable atherosclerotic patients with previous myocardial infarction was three times as much as in patients without previous myocardial infarction (137, 138). Similarly, in the ODYSSEY OUTCOMES study, the use of alirocumab in addition to statin treatment significantly reduced the risk of MACEs and death in patients with ACS, which is more significant in patients with previous myocardial infarction (139).

The level of plasma PCSK9 is positively correlated with hs-CRP in myocardial infarction patients (140). Higher plasma levels of PCSK9 and hs-CRP lead to an earlier decline in left ventricular ejection fraction in myocardial infarction patients, further increasing the risk of myocardial infarction-induced heart failure (HF) and even death (140). In C57BL6/J WT mice with myocardial infarction, PCSK9 promotes the polarization of M1-type macrophages and inhibits the polarization of M2-type macrophages by activating the TLR4/MyD88/NF-κB signaling pathway, leading to the significant inflammatory response, increased infarct size, and excessive impairment of cardiac function (43).

In addition, CD36-mediated thrombosis and NLRP3 inflammasome-mediated autophagy are both associated with the effect of PCSK9 on myocardial infarction prognosis (26, 141). Regardless of the specific underlying mechanism, upregulated PCSK9 is unfavorable to the recovery of myocardial infarction.

## 5. Summary and clinical implications

As the number of patients with ASCVDs increases, many evidences show that PCSK9 has become a new promising therapeutic target for patients who are intolerant of statins or who fail to achieve the plasma lipid goal after treatment with a maximum dose of statins (10). In general, PCSK9 affects homeostasis *in vivo* through multiple functional mechanisms, including regulating lipid metabolism, promoting the immune response, promoting platelet activation, promoting thrombosis, promoting apoptosis, promoting pyroptosis, and inhibiting autophagy. PCSK9 promotes the progression of ASCVDs *via* various mechanisms, and it has been confirmed that PCSK9 inhibitors effectively improve the prognosis of patients with ASCVDs (10–15).

## 6. Perspectives and directions for future research

Based on the existing researches on PCSK9 and cardiovascular diseases, we propose several perspectives and directions for future research. First, many studies regarding PCSK9 and immunity in cardiovascular diseases only detected changes in immune cell subsets, and a few studies have demonstrated that the above functions of PCSK9 are mediated by classical pathways, such as TLR4/NF-kB and NLRP3 (27, 43). Thus, it is meaningful to determine whether other pathways associated with inflammation are involved, such as cGAS-STING pathway, Notch pathway et al. (142, 143). Whether PCSK9 regulates the transformation of immune cells subsets by altering their metabolic activity? Which kind of metabolic activity is altered most dramatically? Which kind of immune cells subset is most sensitive to the metabolic effects of PCSK9? These can be further studied. Second, because PCSK9 and CD8+ T cells accumulate in the ischemic heart and the effect of PCSK9 on CD8+ T cells in the tumor microenvironment is already clear, will their cross-talk in the cardiac microenvironment be different from the former (90)? What factors account for this difference? Third, whether PCSK9 inhibitors are useful in improving the prognosis of patient with heart failure (HF) remains controversial. For example, in the multicenter, prospective, observational Biology Study to Tailored Treatment in Chronic Heart Failure (BIOSTAT-CHF) study, the rates of all-cause mortality and the composite of mortality or unscheduled hospitalizations due to HF had a positive association with plasma level of PCSK9 and a negative association with the level of LDLR in HF patients (144). In the ODYSSEY OUTCOMES study, however, it was found that although alirocumab reduced plasma cholesterol levels to the same extent, it did not lower the incidence of MACEs, death, or unscheduled hospitalizations for HF in ACS patients with previous HF (145, 146). The inconsistency among the above findings may be related to the heterogeneity of the enrolled patient population with respect to the cause or severity of the heart injury, or the usage and dosage of PCSK9 inhibitors. Therefore, according to these influencing factors, we can divide the enrolled patients into different subgroups before carrying out our research. Finally, it is valuable to investigate whether PCSK9 inhibitors can improve the prognosis of other common cardiovascular diseases.
